# Effect of probiotics on humoral responses to COVID-19 vaccination in older adults: a randomized, placebo-controlled trial (PIRATES-COV study)

**DOI:** 10.1080/19490976.2026.2699456

**Published:** 2026-07-28

**Authors:** Jean-Charles Pasquier, Nils Chaillet, Mélanie Plourde, Guy Boivin, Marie-Ève Hamelin, Hugues Allard-Chamard, Alain Piché, Geneviève Baron, Isabelle Laforest-Lapointe, Mélissa Généreux, Benoît Mâsse, Julie Robitaille, Jean-François Beaulieu, Tamas Fülöp, Sarah Bilodeau, Sheela Ramanathan

**Affiliations:** a Centre de recherche du Centre hospitalier de l’Université de Montréal, Montreal, Quebec, Canada; b Centre hospitalier de l’Université de Montréal (CHUM), Montreal, Quebec, Canada; c Université de Montréal, Montreal, Quebec, Canada; d Université Laval, Quebec City, Quebec, Canada; e Université de Sherbrooke, Sherbrooke, Quebec, Canada; f Centre hospitalier universitaire Sainte-Justine (CHU Sainte-Justine), Montreal, Quebec, Canada

**Keywords:** COVID-19, vaccination, immunity, clinical trial, aging, probiotic

## Abstract

**Background:**

Although COVID-19 vaccination has reduced morbidity and mortality, adults aged 65 y old and older are at high risk of complications from COVID-19. The evidence suggests that probiotics may enhance immune responses when co-administered with influenza vaccination. However, few studies have evaluated the potential benefit of probiotic supplementation in this context in this high-risk population.

**Material:**

This study was a randomized controlled trial recruiting adults between 65 and 89 y of age living in Quebec, Canada, who received an mRNA booster (Pfizer-BioNTech or Moderna). Probiotic supplementation (*Lacticaseibacillus rhamnosus* Rosell^®^-11 and *Lacticaseibacillus paracasei* Rosell^®^-215, 1x 6 x 10^9^CFU per capsule) versus placebo was administered from 15 d pre- to 15 d post-vaccination. Participants provided dried blood spot samples at baseline, at 3- and 6-month post-vaccination. The primary outcome was the proportion of participants without detectable anti-S1-receptor binding domain (anti-S1-RBD) antibodies at 6 months post-vaccination. Secondary outcomes included longitudinal anti-S1-RBD and anti-nucleocapsid (anti-*N*) antibody responses across the three timepoints.

**Results:**

592 adults were enrolled. At 6 months post-vaccination, anti-S1-RBD antibodies were comparable between groups, and the percentage of participants with undetectable antibody response in the placebo and probiotic groups (1.2% vs 1.6%) was comparable. Anti-*N* seropositivity was lower in the probiotic group at 6 months (OR 0.6, 95% CI 0.4–0.9; *p* = 0.02) and marginally lower at 3 months after adjustment. Self-reported COVID-19 infection incidence was 16.5% overall (19.1% placebo vs 13.9% probiotic; *p* = 0.134).

**Conclusion:**

Probiotic supplementation administered around COVID-19 vaccination did not change the proportion of seronegative participants for anti-S1-RBD antibodies at 6 months. However, in the probiotic group, anti-*N* antibody levels were lower, while self-reported COVID-19 infections also tended to be reduced, although this difference was not statistically significant. They may be consistent with earlier control of infection and reduced exposure to the nucleocapsid antigen, but this interpretation remains speculative.

**Trial registration number:**

#NCT05195151.

## Introduction

The global pandemic of Coronavirus Disease 2019 (COVID-19), caused by Severe Acute Respiratory Syndrome Coronavirus 2 (SARS-CoV-2), resulted in high mortality, severe strain on healthcare systems, and major socio-economic impacts.^1^ Adults over 65 y old have been disproportionately affected by COVID-19, exhibiting markedly higher rates of severe disease, hospitalization, and mortality than younger populations.^2^ This heightened vulnerability is largely attributed to age-related decline in both innate and adaptive immune functions, coupled with a high prevalence of comorbidities.^3^

Unprecedented global scientific mobilization enabled the rapid development and deployment of COVID-19 vaccines, which drastically reduced the morbidity and mortality associated with SARS-CoV-2 infection.^4^ Among the vaccines developed, the Pfizer–BioNTech (BNT162b2) and Moderna (mRNA-1273) vaccines, both encoding the genetic information for the SARS-CoV-2 spike protein, were administered extensively in Canada.^5^ ^,^ ^6^ Large phase III clinical trials confirmed their high efficacy in preventing symptomatic and severe COVID-19. However, interindividual variability in vaccine-induced immune responses, the occurrence of breakthrough infections, and the duration of protection remain major concerns that warrant ongoing investigation.^7^

Vaccination remains highly effective at preventing severe COVID-19 and reducing mortality in older adults; however, responses in this age group exhibit distinct clinical and immunological features. Older adults more frequently experience local and systemic reactogenicity, and vaccine-induced protection wanes more rapidly.^8^ ^,^ ^9^ A growing body of clinical and preclinical evidence indicates that gut microbiota could be a major determinant of interindividual variability in vaccine responses.^10^ Probiotics are live microorganisms that, when administered in adequate amounts, may enhance vaccine immunogenicity.^11^ ^,^ ^12^ Meta-analyses of randomized controlled trials of influenza vaccine trials suggest modest improvements in humoral response with probiotic supplementation.^11^ Although mechanisms remain incompletely defined, the hypothesized pathways include heightened innate immune priming, modulation of adaptive responses via microbially derived metabolites, and broader antigen cross-reactivity.^13^

In the selection of the strains for probiotics capsules, the researchers were constrained to proceed without dedicated preclinical studies because of the pandemic context. The two strains selected were based on a targeted review of the literature, triangulating evidence from three domains: probiotics and COVID-19,^14^ probiotics for older adults,^15^ ^,^ ^16^ and probiotics as vaccine adjuvants.^12^ ^,^ ^17^ ^,^ ^18^ *Lacticaseibacillus rhamnosus* R0011 (8 × 10^9^ CFU/capsule) emerged as the strain with the most consistent evidence of benefit alongside influenza vaccination, was highlighted in candidate gene–COVID association networks, and is among the most extensively used strains in clinical research.^12^ ^,^ ^17^ ^,^ ^18^ *Lacticaseibacillus casei* R0215 (8 × 10^9^ CFU/capsule) has likewise been repeatedly reported for its immunoregulatory properties in both preclinical and clinical studies.^19-21^ In the emergency context of the pandemic, selection of the investigational product was also guided by a well-documented safety record of the strains in humans and considered the availability of a standardized, traceable product with CFU content and stability compatible with the logistical constraints of a clinical trial.

Serologic measures are used to assess the immune response after vaccination. In the context of a novel pandemic with unpredictable fluctuations in disease incidence, it provides a means of monitoring vaccine performance without relying solely on clinical epidemiologic data. By combining different markers, serologic surveillance can also help distinguish vaccine-induced immunity from immunity acquired through natural infection, which in some settings – such as COVID-19 – may be asymptomatic.^22^

Given the high vaccination coverage among older adults and the low reported rate of adverse events associated with probiotic use, even modest improvements in vaccine responses could translate into meaningful public health gains. We hypothesized that administering probiotics concomitantly with COVID-19 mRNA vaccination would enhance humoral immunogenicity during the first 6 months after vaccination, as would be evidenced by higher anti-S1-receptor-binding domain (Anti-S1-RBD)/anti-spike antibody titers. Anti-nucleocapsid (anti-*N*) antibodies were monitored to compare the occurrence of recurrent SARS-CoV-2 infections and also serve as an indirect marker of viral replication.

## Methods

### Study design

This randomized, double-blinded, placebo-controlled trial using intention-to-treat analysis was conducted at the *Centre de recherche du Centre Hospitalier Universitaire de Sherbrooke* (*CR-CHUS*), in Sherbrooke, Quebec, Canada. The primary objective was to reduce the percentage of adults between 65 and 89 y old presenting no anti-S1-RBD antibodies at 6 months after the booster vaccine dose.

To test our hypothesis, the intervention consisted of an oral probiotic supplement or an inert placebo, and participants’ immune response was assessed for up to 6 months following COVID-19 vaccination. The study was registered at an online public clinical trial registry (see abstract), and the protocol has been published.^23^

### Inclusion/exclusion criteria

The study recruited male and female adults aged 65–89 y, living at home or in an independent-living retirement home in the province of Quebec, who wished to receive the subsequent government-recommended COVID-19 mRNA vaccine booster dose. Candidates were required to have already received three vaccine doses (Pfizer-BioNTech or Moderna), the most recent dose being a minimum of five months before inclusion. The participants were also required to speak French and/or English and to have a home telephone and/or internet access. The cognitive ability to participate in the study was assessed using the Functional Activities Questionnaire (FAQ),^24^ excluding those with scores ≥ 9. Other exclusion criteria were an allergy to any of the product ingredients (soy, lactose, etc.), chronic immunodeficiency or immunosuppression (e.g., concurrent cancer chemotherapy), active treatment for intestinal disorders, and use of probiotics or antibiotic treatment at the date of inclusion. Individuals with a serious condition precluding safe participation in the study to the end of the study were also excluded, as well as those who had contracted COVID-19 in the past three months before the next booster shot.

Recruitment strategies were defined in detail in the protocol publication.^23^ Eligibility screening was completed online or over the phone. Depending on participants’ computer skills, an electronic consent form was signed digitally, via REDCap, or orally registered and signed on a paper form.

### Randomization

Randomization was performed using permuted blocks of size 4 and 6, stratified by age (65–79 y, 80–89 y) and sex (male, female), less than 30 d before the initiation of the investigational product. The order of blocks was randomized. Randomization sequences were generated in SAS using the PLAN procedure.^25^

Randomization was programmed by the Applied Clinical Research Unit of the CHU Sainte-Justine Research Centre and performed online using REDCap. To ensure a double-blind study, neither the participants, research team members, partners, nor affiliated laboratories were informed of participants’ group allocation. Research pharmacists at CR-CHUS prepared the study products (probiotics or placebo) as per REDCap blinding requirements.

### Intervention

Participants were given an oral probiotic dietary supplement containing two bacterial strains, *Lacticaseibacillus rhamnosus* Rosell^®^-11 and *Lacticaseibacillus paracasei* Rosell^®^-215 for a total of 6 × 10^^9^ CFU/capsule, or an inert placebo from Lallemand Health Solutions. The participants were instructed to take one capsule per day for a 30-d period, beginning 15 d before receiving their COVID-19 booster shot. Compliance was assessed through participant completion of a logbook once a day.

### Assessments

Each participant received a home kit including the probiotic or placebo capsules, a blood sample collection kit with step-by-step instructions, and a COVID-19 rapid test to be used should any symptoms appear. A first dried blood spot sample was collected at baseline, between randomization and the initiation of the investigational product, using a finger-prick test on two blotter papers (5 × 2 drops of blood deposited, see Appendix D). If the participant was unable to take their samples, an online tutorial and telephone support were provided, or, in some cases, a nurse assisted them at home. Blood spot samples were also taken at 3- and 6-month post-vaccination. All dried blood samples were sent by courier to CR-CHUS immediately once it was done and then transferred to Guy Boivin’s laboratory at *Université Laval*, Quebec, Canada for analysis.

Levels of anti-S1-RBD and anti-*N* antibodies were determined by colorimetric enzyme-linked immunosorbent assays (ELISA) on the blood samples. For blood elution, 2x 6 mm punches were performed for each sample and placed in a 96-well plate. A total of 275 µl per well of elution buffer was added, and the plate was centrifuged at 2000xg for 1 min before resting at room temperature overnight. The next day, the plates were agitated for 30  sec at 700  rpm and centrifuged at 2000xg for 1 min. Eluates were then used for ELISA. The positivity threshold was defined based on the mean optical density (colorimetric reaction on blotting paper) of negative serum + 3 standard deviations. A semi-quantitative variable was available, as there is no standard curve or unit to give a precise number. The value was represented as a ratio of the optical density obtained for the negative controls.

As soon as a participant reported flu-like symptoms to the research team, the participant carried out the COVID-19 home test and was asked to report the result to the research team. Alla COVID-19 infection reportedduring the study period were confirmed by a home test or PCR, and the participant completed a FLU-PRO Plus questionnaire describing their symptoms.^26^

After their COVID-19 booster dose, local and systemic side effects were collected once per week for two weeks.^27^ Expected side effects of the oral probiotics included bloating, intestinal irritation, and/or softer stool than usual. Reports of adverse events were collected once a month by asking participants about changes in their health. The participants were also invited to voluntarily report any changes by directly contacting the research team.

### Outcome measures

The primary outcome measure is a dichotomous measure of anti-S1-RBD antibodies (detectable or undetectable) and was determined by a validated ELISA test (95% sensitivity and 100% specificity). The anti-*N* antibodies were determined using the same procedure as for the dichotomous anti-S1-RBD antibody level (90% sensitivity and 95% specificity). For both antibodies, continuous variables representing semi-quantitative measures were also available.

### Sample size calculation

We calculated the sample size to discern an effect size of 33% reduction in the number of participants presenting undetectable levels of anti-S1-RBD antibodies at 6 months post-vaccination. With an expected undetectable antibody level in 30% of the placebo group and therefore 20% in the intervention group, an estimated 584 participants would provide 80% power and a two-tailed alpha of 5%. We thus determined that with an estimated 15% attrition rate, the study would require 688 participants.

### Statistical analysis

The analysis was performed according to the intention-to-treat principle. No interim analysis was performed. The intervention and placebo groups were compared at baseline for sociodemographic and clinical characteristics. The results for continuous variables were presented as means  ±  standard deviations (SD) or as geometric means and 95% confidence intervals (CI). Results for categorical variables were presented as *n* (%). Statistical models were carried out separately for the three post-vaccination timepoints: baseline, 3- and 6-month. All models were adjusted for age and sex. Logistic regression models were used when outcomes were considered binary (detection of antibodies versus no detection). Firth logistic regression models were used when there was complete separation in the data. Linear regression models were used for the anti-S1-RBD antibody outcome treated as a continuous variable. For the anti-*N* antibody outcome treated as a continuous variable, linear regression models were not appropriate, as the outcome for most of participants was a value of zero, and the skewed distribution led to a violation of the normality assumption of residuals. In this case, a Tweedie regression model was used, as this model is designed for variables that are continuous but include many zeros. Repeated measures models were then carried out using each of the three post-vaccination timepoints. The models were adjusted for age and sex and included interaction terms between time and treatment group. Generalized estimating equations (GEE) models were applied, with a logistic link function for binary outcomes, and with a Gaussian family using an identity link function for the continuous anti-S1-RBD antibody outcome. For the anti-*N* antibody outcome treated as a continuous value, the repeated measures model used was a generalized linear mixed model (GLMM) with a Tweedie distribution and log link. The phi coefficient was used to assess the association between self-reported COVID-19 infection and anti-*N* antibody outcome.

## Ethics

The study was approved by the Research Ethics Board of the *Centre Intégré Universitaire de Santé et des Services Sociaux de l’Estrie–Centre Hospitalier Universitaire de Sherbrooke* (#MP-31-2022-4498) and the *CHU de Québec-Université Laval* (# MEO-31-2022-6278). The study followed national and international regulations on participant privacy and rights. Written informed consent was obtained for all participants for their participation in the study and publication of the results. Owing to the level of involvement required during the study (e.g., self-collection of blood and stool samples), financial compensation was offered to each participant.

## Results

### Participants

**Figure 1.**
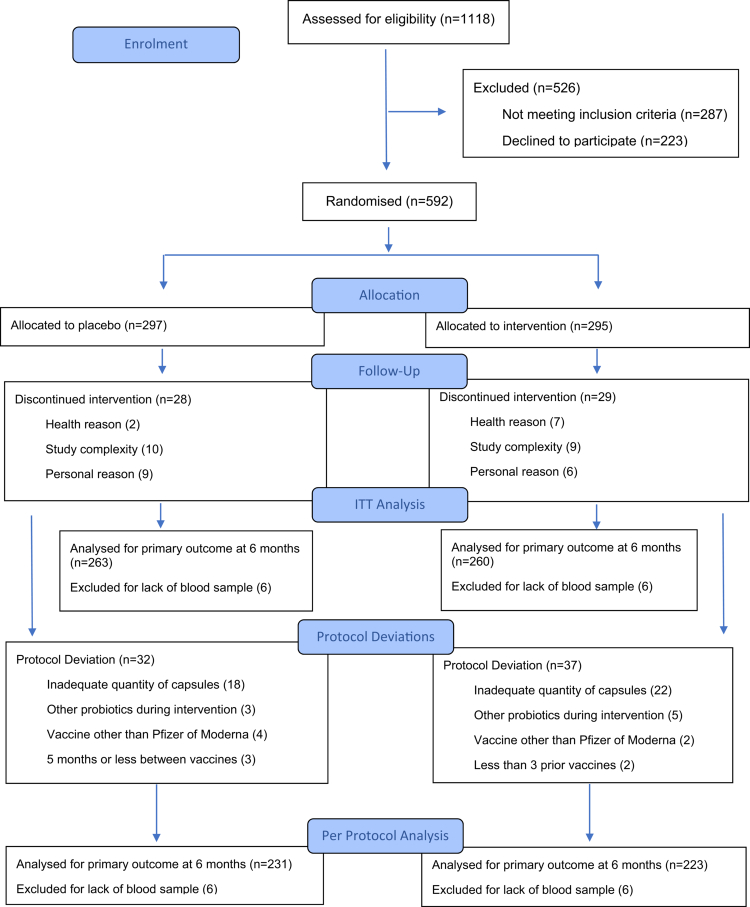
CONSORT 2025 flow diagram.

Between November 2022 and January 2024, 592 participants (273 men and 319 women) were consecutively enrolled in the study. A total of 530 participants were recruited in the 65–79 age group and 62 in the 80–89 age group. An intention-to-treat analysis for anti-S1-RBD antibodies results collected at 6 months was carried out for 523 participants and for 454 compliant participants for per-protocol analysis (see Figure 1). The participant characteristics were comparable between groups (see Table 1). The whole analysis was conducted according to the intention-to-treat principle, and the per-protocol analysis revealed no differences (see Supplementary material, Tables B and C).

**Table 1.** PIRATES-COV study.

Characteristics at baseline	Control (Placebo) *n* = 297	Intervention (Probiotics) *n* = 295	*p*-value
**Age (years)**			
65–79	266 (89.6%)	264 (89.5%)	1,00
80–89	31 (10.4%)	31 (10.5%)	
**Sex**			
Male	137 (46.1%)	136 (46.1%)	1,00
Female	160 (53.9%)	159 (53.9%)	
**Body Mass Index (BMI)**			
Mean (SD)	27.1 (4.6)	26.9 (5.3)	0,78
Median (min, max)	26.9 [17.7, 44.8]	26.4 [13.8, 53.0]	
Missing	12 (4.0%)	8 (2.7%)	
**Born in Canada**	272 (90.4%)	262 (90.3%)	0,60
**Caucasian ethnic group**	277 (93.3%)	280 (94.9%)	
**Employment**			
Retired	266 (91.7%)	269 (92.8%)	0,76
Worker	24 (8.3%)	21 (7.2%)	
**Education**			
High school or less	58 (20.1%)	76 (26.6%)	0,18
Cegep College or trade diploma	99 (34.3%)	86 (30.1%)	
University degree	132 (45.7%)	124 (43.4%)	
**Household income**			
Less than 49 999$	105 (40.7%)	107 (40.7%)	1,00
50 000$ and more	153 (59.3%)	156 (59.3%)	
**Smoking**			
Never	119 (41.2%)	117 (40.9%)	0,84
Previously	156 (54.0%)	152 (53.1%)	
Currently	14 (4.8%)	17 (5.9%)	
**Any COVID-19 infection in the past**	143 (49.3%)	126 (43.4%)	0,18
**Number of vaccines before booster**			
3 vaccines or less	198 (68.3%)	194 (66.9%)	0,79
more than 3 vaccines	92 (31.7%)	96 (33.1%)	

All data are shown as *n* (%), unless otherwise indicated. No statistical differences were observed between groups.

#### Primary outcome

At 6-months post-vaccination, the proportion of participants in the placebo and probiotic groups with no detectable anti-S1-RBD antibodies was similar, with 1.2% (*n* = 3) and 1.6% (*n* = 4), respectively (see Table 2). Given the low proportion of participants with undetectable anti-S1-RBD antibodies, the planned analyses, including GEE, were not performed.

**Table 2.** Linear models for Anti-S1-RBD and Anti-*N* antibodies at baseline, 3- and 6-months post-vaccination, by group.

*Baseline*	Placebo (*n* = 266)	Probiotics (*n* = 264)	
**Anti-S1-RBD**			
Detected, *n* (%)	265 (99.6%)	261 (98.9%)	*p* = 0.34
Mean (SD)	10.1 (6.1)	10.4 (6.0)	*p* = 0.59
**Anti-N**			
Detected, *n* (%)	97 (36.5%)	94 (35.6%)	*p* = 0.83
Mean (SD)	1.1 (2.1)	1.0 (1.9)	*p* = 0.39
***3 Months***	**Placebo** (*n* = 256)	**Probiotics** (*n* = 259)	
**Anti-S1-RBD**			
Detected, *n* (%)	256 (100%)	258 (99.6%)	*p* = 0.47
Mean (SD)	12.0 (6.1)	11.2 (6.1)	*p* = 0.13
**Anti-N**			
Detected, *n* (%)	97 (37.9%)	78 (30.1%)	*p* = 0.06
Mean (SD)	1.6 (2.9)	1.1 (2.1)	*p* = 0.046
***6 Months***	**Placebo** (*n* = 263)	**Probiotics** (*n* = 260)	
**Anti-S1-RBD**			
Detected, *n* (%)	260 (98.9%)	256 (98.5%)	*p* = 0.69
Mean (SD)	10.6 (6.5)	10.1 (6.3)	*p* = 0.34
**Anti-N**			
Detected, *n* (%)	113 (43.0%)	85 (32.7%)	*p* = 0.02
Mean (SD)	1.8 (3.1)	1.2 (2.3)	*p* = 0.01

All analyses were adjusted for randomization criteria (age group and sex).

#### Secondary outcomes

Table 2 shows the distribution of antibody levels of anti-S1-RBD as a continuous variable at each time point. In linear models estimated separately at each time point, no differences were detected between the placebo and probiotic groups. Repeated-measures GEE analysis including a time × group interaction revealed no between-group differences in the evolution of anti-S1-RBD antibodies at either 3 months (*β* = -0.9 [-1.8, 0.1], *p* = 0.09) or 6 months (*β* = -0.8 [-1.9, 0.3], *p* = 0.16).

At baseline, anti-*N* antibodies were detected in 36% of participants. At 3 months, the difference between the proportion of participants with anti-*N* antibodies in the probiotic group was marginally similar after adjusting for age and sex (*p* = 0.06, see Table 2). At 6 months, the probiotic group had significantly lower odds of anti-*N* seropositivity than the placebo group (OR = 0.6, 95% CI 0.4–0.9; *p* = 0.02). Females had higher odds than males to have circulating anti-*N* antibodies (OR = 1.7, 95% CI 1.1–2.5; *p* = 0.01).

After adjusting for age and sex, the probiotic group exhibited significantly lower levels of anti-*N* antibody titers at 3 and 6 months than the placebo group (*p* = 0.046 and *p* = 0.01; see Table 2 and Appendix B). In the repeated measures Tweedie GLMM, only anti-*N* antibody titers were significantly higher at 3 and 6 months than at baseline for overall participants (see Table 3).

**Table 3.** Tweedie generalized linear mixed model for repeated measures for Anti-*N* at baseline, 3- and 6-months post-vaccination.

	MR	95% CI	*p*
**Time (3 months)**	1.3	[1.1, 1.6]	0.003*
**Time (6 months)**	1.5	[1.3, 1.9]	<0.0001*
**Group (Probiotics)**	0,8	[0.5, 1.1]	0.206
**Sex (female)**	1.3	[1.0, 1.9]	0.094
**Age group (80–89 y)**	1.1	[0.6, 1.8]	0.865
**Time (3 months) x Group (Probiotics)**	0.8	[0.6, 1.1]	0.167
**Time (6 months) x Group (Probiotics)**	0.8	[0.6, 1.1]	0.107

*as *p*<0.05 is significant.

For anti-*N* antibodies as a binary variable, a GEE model with a binomial family and a logit link function was used. The interaction term between the two groups showed no difference at 3 months (OR = 0.79 [0.54, 1.17], *p* = 0.237) and at 6 months (OR = 0.73 [0.49, 1.09], *p* = 0.125). The level of antibodies-*N* was analyzed as a continuous variable using a generalized linear mixed model with a Tweedie distribution and a log link. Although no main effect of the study group was detected. The 3-month × probiotic interaction (MR = 0.8 [0.6, 1.1], *p* = 0.167) and the 6-month × probiotic interaction (MR = 0.8; 95% CI, 0.6–1.1; *p* = 0.107) were not statistically different.

**Figure 2.**
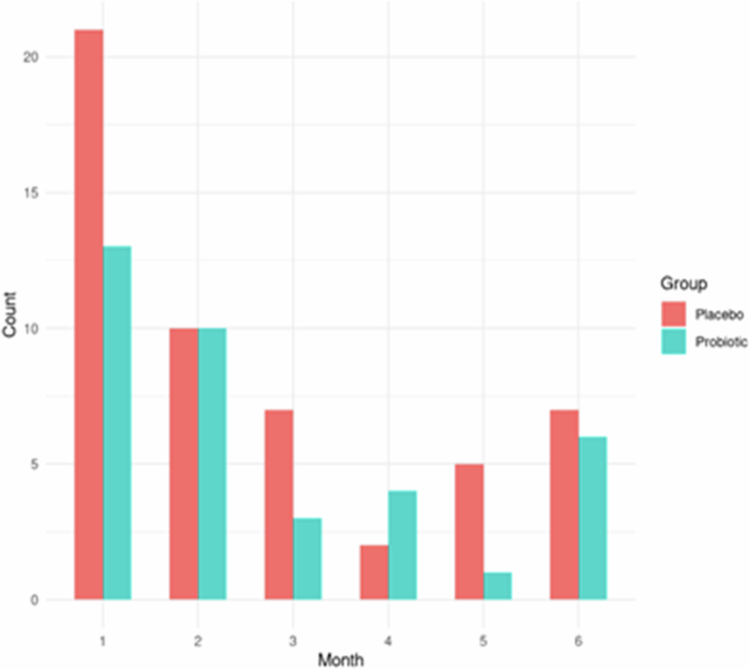
Number of participants infected by COVID-19 for each month, by group.

Of 539 participants, 89 (16.5%) self-reported a COVID-19 infection during follow-up (see Table 4). The incidence was 13.9% in the probiotic group (37/267) versus 19.1% in the placebo group (52/272; *p* = 0.134). Figure 2 shows the number of participants reporting a COVID-19 infection every month, by group. None of the 27 hospitalizations that occurred during the trial (16 in the placebo group; 12 in the probiotic group) were related to COVID-19 complications.

**Table 4.** Self-reported COVID-19 infections during the trial, by group.

	Placebo *n* = 272	Probiotics *n* = 267	Overall *n* = 539
**No COVID-19 infection**	220 (80.9%)	230 (86.1%)	450 (83.5%)
**COVID-19 infection**	52 (19.1%)	37 (13.9%)	89 (16.5%)

All data are shown as *n* (%), there is no significant difference between groups, *p* = 0.13, adjusted for age group and sex.

Self-reported COVID-19 infections were correlated with anti-*N* antibody positivity by dividing the follow-up into two periods: months 1–3, linked to anti-*N* levels at 3 months, and months 4–6, linked to anti-*N* levels at 6 months. A strong association was observed between anti-*N* positivity and the reported COVID-19 infection, see Table 5.

**Table 5.** Association between self-reported COVID-19 infection and anti-*N* antibody.

Between 1 and 3 months			
	**No COVID-19** (*n* = 459)	**Self-reported COVID-19** (*n* = 55)	***p*-value**

**No Detected Anti-*N* Antibodies**	320 (69.7%)	19 (34.5%)	< 0.0001
**Detected Anti-*N* antibodies**	139 (30.3%)	36 (65.5%)	
**Between 4 and 6 months**			
	**No COVID-19** (*n* = 485)	**Self-reported COVID-19** (*n* = 37)	***p*-value**

**No Detected Anti-*N* antibodies**	315 (64.9%)	9 (24.3%)	< 0.0001
**Detected Anti-*N* antibodies**	170 (35.1%)	28 (75.7%)	
**Over 6 months**			
	**No COVID-19** (*n* = 440)	**Self-reported COVID-19** (*n* = 81)	***p*-value**

**No detected Anti-*N* antibodies**	295 (67.0%)	28 (34.6%)	< 0.0001
**Detected Anti-*N* antibodies**	145 (33.0%)	53 (65.4%)	

**Figure 3.**
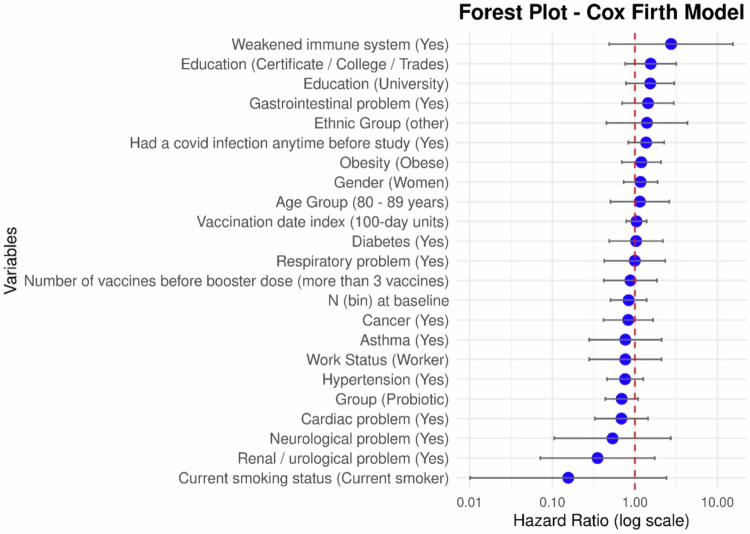
Risk factors of developing COVID-19 infection.

Risk factors of contracting COVID-19 infection were analyzed using a Cox Firth-penalized logistic regression with stepwise selection. No factors were linked to higher odds of infection at 6 months, including assignment to probiotic or placebo group (see Figure 3).

#### Safety profile and adverse events

Vaccine-related adverse events, including local (injection-site pain or redness) and systemic (fever, fatigue, headache, myalgia, gastrointestinal symptoms, and flu-like illness) reactions, were similar in both groups (see Table 6). The adverse events monitored throughout the study are presented in Table 7. The number of subjects presenting at least one adverse event other than COVID-19 infection was not different between the placebo group (164/297; 55.2%) and the probiotic group (160/295; 54.2%) (*p* = 0.88). The most frequently reported adverse events were cold, constipation, and diarrhea. Twenty-six serious adverse events were reported during the study, 13 (4.4%) in the placebo group and 13 (4.4%) in the probiotic group. None were considered related or possibly related to the investigational product.

**Table 6.** Self-reported COVID-19 vaccine side effects, by group.

Vaccine side effect	Placebo *n* = 266 (week 1) *n* = 264 (week 2)	Probiotics *n* = 268 (week 1) *n* = 266 (week 2)
**Local reaction** ^**a**^		
No	138 (51.9%)	145 (54.1%)
Redness at the injection site	120 (45.1%)	117 (43.7%)
Redness over the shoulder or below the elbow	8 (3.0%)	6 (2.2%)
**New or worsened health issue**		
Week 1	16 (6.0%)	21 (7.8%)
Week 2	11 (4.2%)	15 (5.6%)
**At least one common sytemic symptom** ^**b**^ **:**		
**Overall List**		
Week 1	127 (47.7%)	116 (43.3%)
Week 2	83 (31.4%)	94 (35.3%)
**General category** ^**c**^		
Week 1	111 (41.7%)	107 (39.9%)
Week 2	72 (27.3%)	81 (30.5%)
**Neurological** ^**d**^		
Week 1	43 (16.2%)	40 (14.9%)
Week 2	33 (12.5%)	34 (12.8%)
**Cardio-respiratory** ^**e**^		
Week 1	32 (12.0%)	24 (9.0%)
Week 2	26 (9.8%)	28 (10.5%)
**Allergic-like** ^**f**^		
Week 1	29 (10.9%)	33 (12.3%)
Week 2	35 (13.3%)	34 (12.8%)

All data are shown as *n* (%), there is no statistical difference between groups. a – All participants were asked about injection site reactions (Redness, pain, or swelling at the injection site/above and below in the immunized arm). b – Only those who indicated a health event that led to work absenteeism, a medical consultation, and/or prevention of daily activities were asked to provide details, and participants could report more than one symptom. c – Unwell (tiredness, weakness, muscle aches, fatigue, or chills), fever, runny nose, GI symptoms (nausea, vomiting, diarrhea, or stomach pain), sore throat, etc. d – weakness or paralysis of the arms or legs/confusion/change in personality/behavior or difficulty with urination or defecation, e – palpitation, breathing difficulty, chest tightness, cough, f – face or lip swelling, eyelid swelling, red eyes, rash or hives, anaphylaxis, coagulation symptoms (symptoms of blood clot or bleeding: swelling/pain in legs).

**Table 7.** Most frequent adverse events reported, by group.

Adverse event	Placebo *n* = 297	Probiotics *n* = 295
Cold	21	13
Constipation	10	10
Diarrhea	7	3
Flu	2	4
Headache	5	1
Stomach Cramps	7	6

## Discussion

In this randomized placebo-controlled trial conducted in adults aged 65–89 y old, probiotic supplementation administered with a COVID-19 vaccine did not change anti-S1-RBD antibody responses at 6 months compared with placebo but was associated with lower anti-*N* antibody levels. A trend toward fewer reported clinical COVID-19 infections was also observed, although this difference did not reach statistical significance. To our knowledge, this is the most comprehensive study to date examining the effects of probiotic supplementation on anti-S1-RBD and anti-*N* antibodies in adults aged 65–89 after COVID-19 vaccination.

In this trial, the prespecified primary outcome, anti-S1-RBD antibody negativity at 6 months, occurred much less frequently than anticipated when the study was designed. This substantially reduced the statistical power to detect between-group differences for the primary endpoint and limited the ability of the trial to determine whether probiotic supplementation reduced the proportion of seronegativity at 6 months post-vaccination.

The prespecified primary outcome was the proportion of participants with undetectable anti-RBD antibodies, used as a pragmatic marker of waning vaccine-induced immunity. Unexpectedly, undetectability remained rare (<2% at all time points), limiting between-group comparisons as described in the statistical analysis plan. When the study was designed, in the context of the pandemic, the prevalence of undetectable anti-spike antibodies after a third vaccine dose was unknown after 6 months. Subsequent evidence indicates that undetectable anti-spike antibodies are rare at six months following a booster, including among adults aged 65–89. Matsuura et al. reported that anti-RBD antibody titers declined markedly over the 6 months following BNT162b2 vaccination, with lower responses in older adults, although antibodies generally remained detectable at 6 months.^28^ In the study by Jolliffe et al., undetectable anti-spike antibodies were rare (4.2%) at 3 months post-vaccination for the overall population, with a higher prevalence among older adults.^29^ In the PIRATES-COV trial, no meaningful differences between groups in semiquantitative anti-RBD antibody levels were observed. Although anti-RBD titers after COVID-19 vaccination are widely reported, few randomized trials have assessed their longitudinal trajectories under probiotic supplementation versus placebo. Forsgård et al. assessed the influence of probiotic supplementation on antibody responses to COVID-19 infection and vaccination (a 6-month intervention with *Limosilactobacillus reuteri* plus vitamin D3).^30^ The vaccination-related data were exploratory and did not allow definitive conclusions on the effect of probiotics. Rodríguez-Blanque et al. randomized 250 frontline health care workers to receive *Loigolactobacillus coryniformis* K8 or placebo for 2 months. Among participants vaccinated during the intervention, anti-spike (S1/S2) IgG levels declined over time but were higher in the probiotic group among those assessed at ≥ 81 d after the first dose.^31^ Fernández-Ferreiro et al. conducted a randomized, double-blind, placebo-controlled trial in 200 nursing home residents aged 60 y and over who received daily *L. coryniformis* K8 for 3 months, starting 10 d after the first mRNA vaccine dose,^32^ suggesting that the probiotics may have contributed to higher titers. No differences between the groups were observed in the antibody levels against SARS-COV-2 spike protein.

In the present study, the proportion of samples with detectable anti-*N* antibodies and the titers were significantly lower in the probiotic group than in the placebo group, with consistent results across both binary and continuous analyses. Anti-*N* antibodies are an indirect marker of the occurrence, intensity, and duration of viral persistence during infection. Factors that reduce peak viral load, shorten the viral replication phase, or accelerate viral clearance decrease nucleocapsid antigen availability and may result in lower anti-*N* titers. It is reasonable to assume that the randomization resulted in the exposure of placebo and probiotic groups to the SARS-CoV-2 virus at comparable levels. We therefore consider it likely that, under comparable exposure, infections in the probiotic group were controlled more rapidly, limiting exposure of the immune system to nucleocapsid protein, thereby resulting in lower anti-*N* seropositivity.

We could hypothesize that, in the context of comparable exposure to SARS-CoV-2, infections occurring in the probiotic group may have been controlled more rapidly, thereby limiting immune-system exposure to the nucleocapsid protein and potentially contributing to lower anti-*N* seropositivity. However, this interpretation should be considered with caution, particularly because probiotics did not show a significant effect on anti-RBD antibody titers.

Two potential mechanisms of action can be involved. First, probiotics may enhance booster-induced anti-vaccine antibody titers, thereby improving vaccine effectiveness. It is well established that vaccine-primed immunity can attenuate early viral dynamics and hasten viral decline/clearance during breakthrough infection, thereby reducing exposure to nucleocapsid antigen and lowering the likelihood and/or magnitude of anti-*N* seroconversion.^33^ ^,^ ^34^ Follmann et al. reported that among vaccinated participants developing COVID-19, anti-*N* seropositivity at a median of 53 d after diagnosis was 40%, compared with 93% among placebo recipients (unvaccinated).^35^ The second mechanism may involve the effect of probiotics on the early course of infection. Some probiotics can modulate innate and mucosal immunity through the gut‒lung axis, promoting earlier control of viral replication.^36^ Reduced exposure to nucleocapsid protein could be compatible with a more modest anti-*N* IgG response, as probiotic supplementation was initiated before and continued after vaccination in our study. Wischmeyer et al., who evaluated *Lacticaseibacillus rhamnosus* GG (LGG) as post-exposure prophylaxis, reported that participants randomized to LGG experienced fewer illness symptoms and a longer time to COVID-19 diagnosis than those receiving a placebo.^37^

Clinically, the higher number of COVID-19 infections observed in the placebo group than in the probiotic group supports a potential effect of the intervention. This imbalance should be interpreted with caution, given the limited number of events and the case-ascertainment procedures. While participants received an antigen self-testing kit at enrollment, reporting and collection of test results were not systematic, which may have contributed to under- or overestimating the number of infections.

### Strengths and limitations

Key strengths of this trial include a prespecified, published protocol with a predefined sampling schedule, standardized laboratory procedures, and a detailed statistical analysis plan. High compliance with the intervention and low loss to follow-up, including at 6 months, further supports the internal validity of the findings. Also, the use of probiotics at the time of vaccination appeared to be a safe approach. Finally, the enrollment of a high-risk population enhances the clinical relevance of the study and addresses a question with direct implications for practice.

The main limitation of the study was the lack, at the time it was designed, of robust data on the expected proportion of participants with undetectable anti-RBD antibodies after a booster dose. This uncertainty undermined the assumptions that informed both the selection of the primary endpoint and the sample size calculation. Additional limitations stem from the fact that serological endpoints provide only an imperfect proxy for clinical protection. Moreover, the use of dried blood spot sampling in an outpatient setting may not fully support standardized quantification, potentially introducing analytical variability. The evolving vaccination landscape and epidemiological context during the trial, subsequent infections resulting from variants, and the degree of cross-reactivity to the vaccine strain amid the urgency of the pandemic response may also have reduced statistical power to detect a modest intervention effect. Finally, the results may not be generalizable beyond the specific strain, dose, and duration of probiotic intake evaluated, and residual bias cannot be excluded given partially self-reported compliance and potentially incomplete ascertainment of incident infection.

The lack of a primary-endpoint effect limits the trial’s clinical implications and, taken in isolation, does not support recommendations for routine probiotic supplementation after vaccination. We are currently assessing humoral and cellular immune responses in a subgroup of 100 participants using whole-blood samples collected at baseline and 6 months post-vaccination. In parallel, analysis of baseline stool samples from all participants will provide a more detailed characterization of gut microbiota features in this older population.

In summary, this double-blind randomized controlled trial, conducted in the context of the pandemic, tested a probiotic supplement as an adjuvant for enhancing COVID-19 immune response to the vaccine in the older adult population. The intervention did not increase anti-RBD antibody responses quantitatively. Anti-*N* antibody levels were significantly lower 6 months post-booster. This pattern suggests a different post-infection trajectory, consistent with earlier control of COVID-19 and reduced exposure to nucleocapsid antigen, possibly reflecting reduced viral replication. These findings should be interpreted with caution and need to be confirmed in future studies. In this setting, and given the generally favorable safety profile of probiotics, even a modest improvement in vaccine responses could translate into a substantial public health benefit in this population, who are more susceptible to infections in general.

## Supplementary Material

Supplementary Material Supp_material20260604_clean.docx

## Data Availability

The participants of this study did not give written consent for their data to be shared publicly, so due to the sensitive nature of the research, supporting data is not available. However, upon request to the corresponding author, some data not related to participants could be shared.
