# Supplementary material for: Effect of probiotics on humoral responses to COVID-19 vaccination in older adults: a randomized, placebo-controlled trial (PIRATES-COV study)
Source: Gut Microbes. 2026 Jul 28;18(1):2699456. doi: 10.1080/19490976.2026.2699456 (PMC13418469; doi:10.1080/19490976.2026.2699456)

Supplementary material

PIRATES-COV study

| Appendix A- Comorbidities at Baseline by Group | |  |
| --- | --- | --- |
|  |  |  |
|  | **Placebo** n=290 | **Probiotics** n=290 |
| **Asthma** |  |  |
| No | 266 (91.7%) | 270 (93.1%) |
| Yes | 24 (8.3%) | 20 (6.9%) |
| **Cancer** |  |  |
| No | 247 (85.2%) | 245 (84.5%) |
| Yes | 43 (14.8%) | 45 (15.5%) |
| **Diabetes** |  |  |
| No | 254 (87.6%) | 248 (85.5% |
| Yes | 36 (12.4%) | 42 (14.5%) |
| **Cardiac Problem** |  |  |
| No | 238 (82.1%) | 245 (84.5%) |
| Yes | 52 (17.9%) | 45 (15.5%) |
| **Respiratory Problem** |  |  |
| No | 258 (89.0%) | 260 (89.7%) |
| Yes | 32 (11.0%) | 30 (10.3%) |
| **Hypertension** |  |  |
| No | 178 (61.4%) | 179 (61.7%) |
| Yes | 112 (38.6%) | 111 (38.3%) |
| **Gastrointestinal Problem** |  |  |
| No | 263 (90.7%) | 265 (91.4%) |
| Yes | 27 (9.3%) | 25 (8.6%) |
| **Neurological Problem** |  |  |
| No | 276 (95.2%) | 280 (96.6%) |
| Yes | 14 (4.8%) | 10 (3.4%) |
| **Urological/Renal Problem** |  |  |
| No | 275 (94.8%) | 275 (94.8%) |
| Yes | 15 (5.2%) | 15 (5.2%) |
| **Weakened Immune System** |  |  |
| No | 287 (99.0%) | 286 (99.0%) |
| Yes | 3 (1.0%) | 3 (1.0%) |
| All data are shown as n (%). There is no statistical difference between groups. | | |
|  | |  |


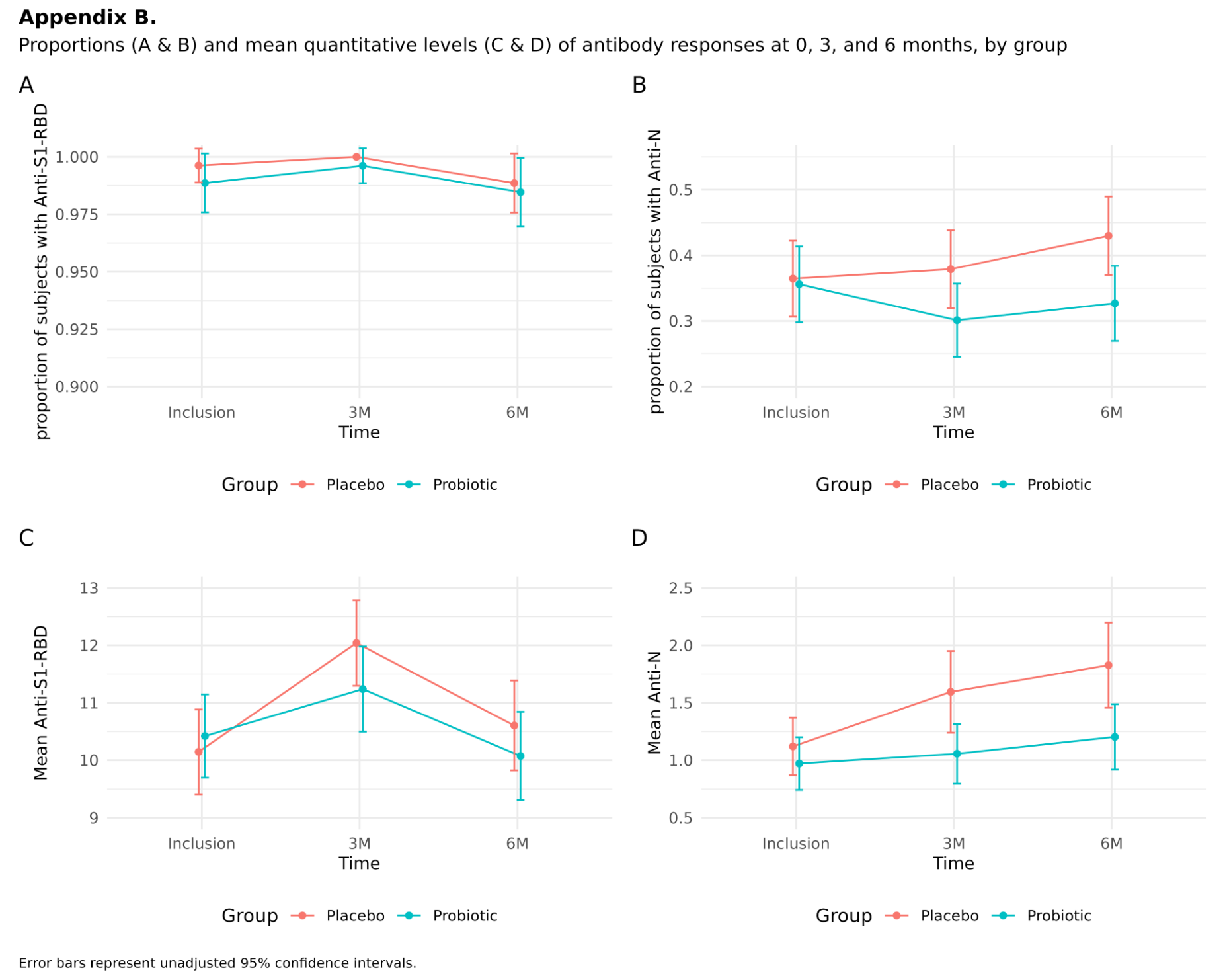


Appendix C- Linear Models for Anti-S1-RBD and Anti-N Antibodies at Baseline, 3- and 6-Months Post-Vaccination, by Group (Per Protocol Analysis)

| ***Baseline*** | **Placebo** (n= 231) | **Probiotics** (n=220) |  |
| --- | --- | --- | --- |
| **Anti-S1-RBD** |  |  |  |
| Detected, n (%) | 231 (100%) | 217 (98.6%) | 0.53* |
| Mean (SD) | 10.2 (6.1) | 10.1 (5.8) | 0.86* |
| **Anti-N** |  |  |  |
| Detected, n (%) | 86 (37.2%) | 77 (35.0%) | 0.62* |
| Mean (SD) | 1.1 (2.1) | 1.0 (2.0) | 0.42* |
| ***3 Months*** | **Placebo** (n= 228) | **Probiotics** (n=223) |  |
| **Anti-S1-RBD** |  |  |  |
| Detected, n (%) | 228 (100%) | 222 (99.6%) | 0.42* |
| Mean (SD) | 12.1 (6.1) | 11.5 (6.1) | 0.30* |
| **Anti-N** |  |  |  |
| Detected, n (%) | 86 (37.7%) | 72 (32.3%) | 0.23* |
| Mean (SD) | 1.6 (3.0) | 1.1 (2.2) | 0.08* |
| ***6 Months*** | **Placebo** (n= 231) | **Probiotics** (n=223) |  |
| **Anti-S1-RBD** |  |  |  |
| Detected, n (%) | 228 (98.7%) | 220 (98.7%) | 0.94* |
| Mean (SD) | 10.9 (6.4) | 10.3 (6.2) | 0.26* |
| **Anti-N** |  |  |  |
| Detected, n (%) | 98 (42.4%) | 79 (35.4%) | 0.14* |
| Mean (SD) | 1.9 (3.1) | 1.3 (2.4) | 0.04* |

*adjusted for age and sex

Appendix D- Repeated Measures Models for Anti-S1-RBD and Anti-N at 3-and 6-months Post-Vaccination (Per Protocol Analysis)

|  | **Time (3 Months) x Group (Probiotics)** | **Time (6 Months) x Group (Probiotics)** |
| --- | --- | --- |
| **Anti-RBD** |  |  |
| Mean (SD) | (-0.5) [-1.6,0.5]; p=0.30 | (-0.7) [-1.8, 0.5]; p=0.26 |
| **Anti-N** |  |  |
| Detected | 0.9 [0.6,1.3]; p=0.52 | 0.8 [0.5,1.3]; p=0.43 |
| Mean (SD) | 0.8 [0.6,1.1]; p=0.23 | 0.8 [0.6,1.1]; p=0.25 |
| all data presented as OR [IC]; p-value | |  |

Appendix E: Blood Spot Sample Realized by a Participant


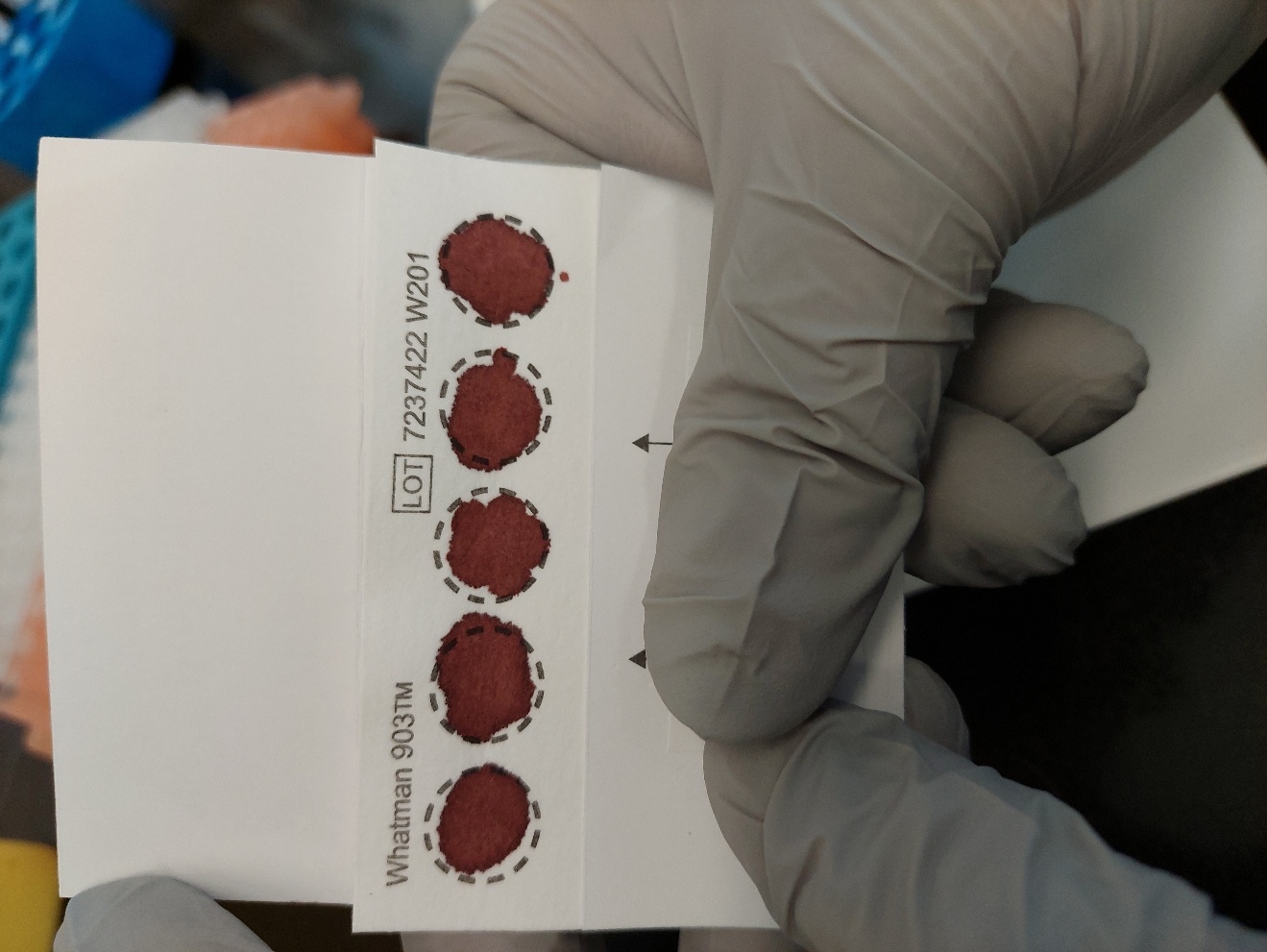

Supplement: Supplementary Material — Supp_material20260604_clean.docx [file KGMI_A_2699456_SM4234.docx]
